# Electrophysiological Substrate and Ablation Outcomes in Atrial Fibrillation With Hypertrophic Cardiomyopathy: A Propensity‐Matched Multicenter Study

**DOI:** 10.1002/joa3.70364

**Published:** 2026-05-23

**Authors:** Umair Abrar, Fazal Ghaffar, Muhammad Zuhaid, Huzaifa Bin Afzaal, Palwasha Abbasi, Muhammad Hasnain Iqbal, Moʻminov Jahongir Zokirjon oʻgʻli, Sifat Ullah Safi

**Affiliations:** ^1^ Orthopaedic and Medical Institute (Pvt) Ltd. Karachi Pakistan; ^2^ King Fahd Military Medical Complex Dhahran Saudi Arabia; ^3^ Warwick Hospital South Warwickshire University NHS Foundation Trust Warwick UK; ^4^ Rai Medical College Sargodha Sargodha Pakistan; ^5^ Suleman Roshan Medical College Tando Adam Sindh Pakistan; ^6^ HBS Medical & Dental College Islamabad Pakistan; ^7^ Ferg‘ona Jamoat Salomatligi Tibbiyot Instituti Fergana Uzbekistan; ^8^ Kabul Medical University Kabul Afghanistan

**Keywords:** atrial fibrillation, catheter ablation, electrophysiological substrate, hypertrophic cardiomyopathy, low‐voltage area, propensity score matching

## Abstract

**Background:**

Atrial fibrillation (AF) is the most common sustained arrhythmia in hypertrophic cardiomyopathy (HCM), yet the intracardiac electrophysiological substrate underlying recurrence following catheter ablation remains poorly characterized. We compared the atrial substrate and ablation outcomes between HCM‐AF patients and propensity‐score‐matched lone AF controls.

**Methods:**

This multicenter retrospective study was conducted across three tertiary centers in Pakistan (2015–2023). Of 841 screened patients, 143 HCM‐AF patients were matched 1:1 with 143 lone AF controls on 14 covariates. Left atrial (LA) electroanatomic voltage mapping, intracardiac electrophysiological variables, and AF inducibility were compared. Primary endpoints were LA low‐voltage area (LVA) burden, AF inducibility, and 12‐month freedom from recurrence post‐ablation.

**Results:**

HCM‐AF patients had greater LVA burden (29.7% ± 17.4% vs. 11.8% ± 9.7%, *p* < 0.001), wider AERP dispersion (41.3 ± 15.7 vs. 18.6 ± 9.4 ms, *p* < 0.001), and higher AF inducibility (81.8% vs. 58.7%, *p* < 0.001). Among 261 ablated patients, 12‐month freedom from recurrence was 51.7% in HCM‐AF versus 73.9% in lone AF (*p* = 0.001). LVA burden (aHR 1.41 per 10% increase), HCM diagnosis (aHR 2.19), non‐paroxysmal AF (aHR 1.88), and AERP dispersion ≥ 30 ms (aHR 1.67) independently predicted recurrence. Latent class analysis identified three EP phenotype clusters with divergent outcomes: 81.7%, 61.4%, and 31.9% recurrence‐free survival (*p* < 0.001). Complication rates were comparable (4.7% vs. 2.2%, *p* = 0.33).

**Conclusion:**

HCM‐AF is associated with a more arrhythmogenic atrial substrate and inferior ablation outcomes compared with lone AF. LVA burden is the strongest predictor of recurrence. EP substrate phenotyping may enable meaningful risk stratification in this population.

## Graphical abstract

Multicentre retrospective propensity‐score‐matched cohort study (*n* = 286; 3 centres, Pakistan; 2015‐2023) comparing electrophysiological substrate and 12‐month ablation outcomes in patients with atrial fibrillation and hypertrophic cardiomyopathy (HCM‐AF, red) versus lone atrial fibrillation (Lone AF, green; 1:1 PSM on 14 covariates).

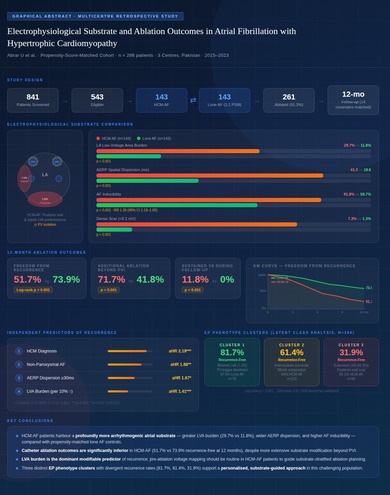

## 1 Introduction

Hypertrophic cardiomyopathy (HCM) is the most prevalent inherited cardiovascular disorder, affecting approximately 1 in 500 individuals worldwide, and remains a leading cause of sudden cardiac death in young adults and a major contributor to heart failure and stroke [1, 2]. Atrial fibrillation (AF) is the most frequently encountered sustained arrhythmia in patients with HCM, with a reported prevalence of 20%–25% and a four‐ to six‐fold higher incidence compared with age‐matched individuals in the general population [3, 4]. The development of AF in HCM portends a significantly worse clinical trajectory, as it is independently associated with an increased risk of thromboembolic stroke, progressive heart failure, functional deterioration, and all‐cause mortality [5, 6].

The pathophysiology of AF in HCM is multifactorial and closely linked to the underlying myopathic process. Left atrial enlargement driven by diastolic dysfunction, elevated left ventricular filling pressures, mitral regurgitation secondary to systolic anterior motion of the mitral valve, and left ventricular outflow tract obstruction collectively create a haemodynamic milieu that promotes progressive left atrial dilatation and structural remodeling [7]. At the tissue level, interstitial and replacement fibrosis—hallmarks of the HCM myocardium—extend into the atrial walls, giving rise to regions of slow conduction, increased anisotropy, and heterogeneous refractoriness that constitute a highly arrhythmogenic substrate [8]. However, detailed intracardiac electrophysiological characterization of this substrate, particularly through high‐density electroanatomic voltage mapping, remains limited.

Catheter ablation has emerged as an established rhythm‐control strategy for symptomatic AF, yet outcomes in patients with HCM are consistently inferior to those observed in structurally normal hearts. Meta‐analytic data indicate that single‐procedure success rates in HCM patients range from only 39%–52%, with a substantially higher need for repeat ablation and continued antiarrhythmic drug therapy compared with non‐HCM controls [9, 10]. These findings underscore the complexity of the atrial substrate in HCM; however, the specific electrophysiological determinants that drive this excess recurrence risk—including left atrial low‐voltage area burden, atrial refractoriness dispersion, and conduction abnormalities—have not been systematically compared between HCM‐AF and lone AF populations using contemporary mapping techniques [11, 12].

In this multicenter retrospective cohort study, we aimed to comprehensively characterize the intracardiac electrophysiological substrate of AF in patients with HCM compared with propensity‐score‐matched lone AF controls. Specifically, we sought to compare left atrial low‐voltage area burden, atrial effective refractory period dispersion, and AF inducibility between the two groups, and to evaluate 12‐month freedom from AF recurrence following catheter ablation. We further aimed to identify independent electrophysiological predictors of AF recurrence and to explore distinct EP substrate phenotypes through unsupervised clustering analysis. These data may inform more refined risk stratification and substrate‐guided ablation strategies in this challenging patient population.

## 2 Methods

### 2.1 Study Design

This was a multicenter, retrospective cohort study conducted at three tertiary electrophysiology centers in Pakistan. Consecutive patients who underwent invasive electrophysiology (EP) study with or without catheter ablation between January 2015 and December 2023 were screened. All centers operate dedicated EP laboratories with three‐dimensional electroanatomic mapping (CARTO 3, Biosense Webster; and/or EnSite Precision, Abbott) and radiofrequency ablation infrastructure. The study was conducted in accordance with the Declaration of Helsinki, with institutional review board approval and waiver of informed consent at each center. This manuscript adheres to the STROBE guidelines [13].

### 2.2 Patient Population

The HCM‐AF group included patients with: (i) confirmed HCM (wall thickness ≥ 15 mm, or ≥ 13 mm in first‐degree relatives) per ESC and AHA/ACC guidelines [14, 15], (ii) documented AF on ECG, Holter, or device electrogram, (iii) index EP study at a participating center, and (iv) age ≥ 18 years with complete EP data. Controls were consecutive lone AF patients with no structural heart disease (wall thickness ≤ 12 mm, LVEF ≥ 55%, no valvular disease or cardiomyopathy). Patients with wall thickness 13–14 mm were excluded from both groups. Exclusion criteria included prior AF ablation, prior cardiac surgery, channelopathy, incomplete documentation, active infection, uncorrected thyroid dysfunction, or electrolyte disturbance.

Controls were matched 1:1 using propensity scores estimated by logistic regression on 14 covariates (age, sex, AF type, LA diameter, LAVI, LVEF, BMI, hypertension, diabetes, CHA_2_DS_2_‐VASc score, heart failure hospitalization, chronic kidney disease, baseline AAD use, and center), with nearest‐neighbor matching (caliper 0.2 SD) without replacement. E/e′ ratio was excluded from the model as a direct consequence of HCM‐related diastolic dysfunction.

### 2.3 EP Study Protocol and Data Collection

Data were extracted by two blinded research associates per center using a standardized form, with discrepancies adjudicated by a senior electrophysiologist. AERP was measured at the HRA, CS proximal, and CS distal using an S1–S2 extrastimulus protocol (drive cycle 600 ms) and defined as the longest coupling interval failing to elicit a propagated response. Per‐patient AERP dispersion was calculated as the maximum minus minimum across three sites. Conduction times (PA interval, P‐wave duration, CS activation time) were measured during sinus rhythm. AF inducibility was assessed via programmed stimulation from the HRA (burst pacing and extrastimulus at 600 ms and 400 ms), with sustained AF (> 30 s) as a positive result; no isoproterenol was used.

### 2.4 Ablation Procedure and Electroanatomic Mapping

Left atrial voltage mapping was performed using CARTO 3 or EnSite Precision (minimum 50 points per patient) during sinus rhythm; LVA was defined as bipolar voltage 0.1–0.5 mV and dense scar as < 0.1 mV [16]. All maps were reviewed centrally by two blinded electrophysiologists. PVI targeting bidirectional block was performed in all patients. Additional ablation (posterior wall isolation, roof line, mitral isthmus line, CTI, CFAE‐guided ablation) was performed at operator discretion based on voltage map findings. Acute success was defined as bidirectional block in all targeted PVs.

### 2.5 Follow‐Up and Monitoring

Oral anticoagulation was continued for ≥ 3 months post‐ablation; subsequent management was at physician discretion. AADs were typically weaned after the 90‐day blanking period. Follow‐up visits occurred at 1, 3, 6, and 12 months with 12‐lead ECG at each visit and 24‐h Holter at 3, 6, and 12 months. ICD interrogation was performed where applicable. All monitoring was reviewed by two blinded cardiologists.

### 2.6 Endpoints

Primary endpoints were: (i) LA LVA burden, (ii) AF inducibility rate, and (iii) 12‐month freedom from AF recurrence (absence of AF/AT/AFL > 30 s after a 90‐day blanking period, per the 2017 HRS/EHRA/ECAS/APHRS/SOLAECE consensus [17]). Arrhythmia events during the blanking period did not count toward the primary endpoint. Secondary endpoints included AERP and conduction parameters, acute PVI success, complication rates, AAD use at 12 months, and all‐cause mortality. All endpoints were prespecified, and recurrence was adjudicated by two blinded cardiologists.

### 2.7 Statistical Analysis

Continuous variables are expressed as mean ± SD or median (IQR); categorical variables as *n* (%). Between‐group comparisons used Student's *t*‐test or Mann–Whitney *U* for continuous and chi‐square or Fisher's exact test for categorical variables. Covariate balance was assessed by SMD (< 0.10 indicating adequacy). Freedom from recurrence was analyzed by Kaplan–Meier with log‐rank testing. Cox proportional hazards regression identified independent predictors of recurrence, with the proportional hazards assumption verified by Schoenfeld residuals. Missing data (5%–20%) were handled by MICE (20 datasets); variables with > 20% missingness were excluded. Prespecified subgroup analyses compared obstructive versus nonobstructive HCM, paroxysmal versus persistent AF, and LVA burden > 10% versus ≤ 10%. With 143 patients per group and a pooled SD of 14%, the study had > 99% power to detect a 15% LVA difference and > 95% power to detect a 20‐percentage‐point difference in recurrence‐free survival (alpha = 0.05). Analyses were performed using SPSS 28.0 and R 4.3.1; *p* < 0.05 was considered significant.

## 3 Results

### 3.1 Patient Enrolment and Baseline Characteristics

**FIGURE 1:**
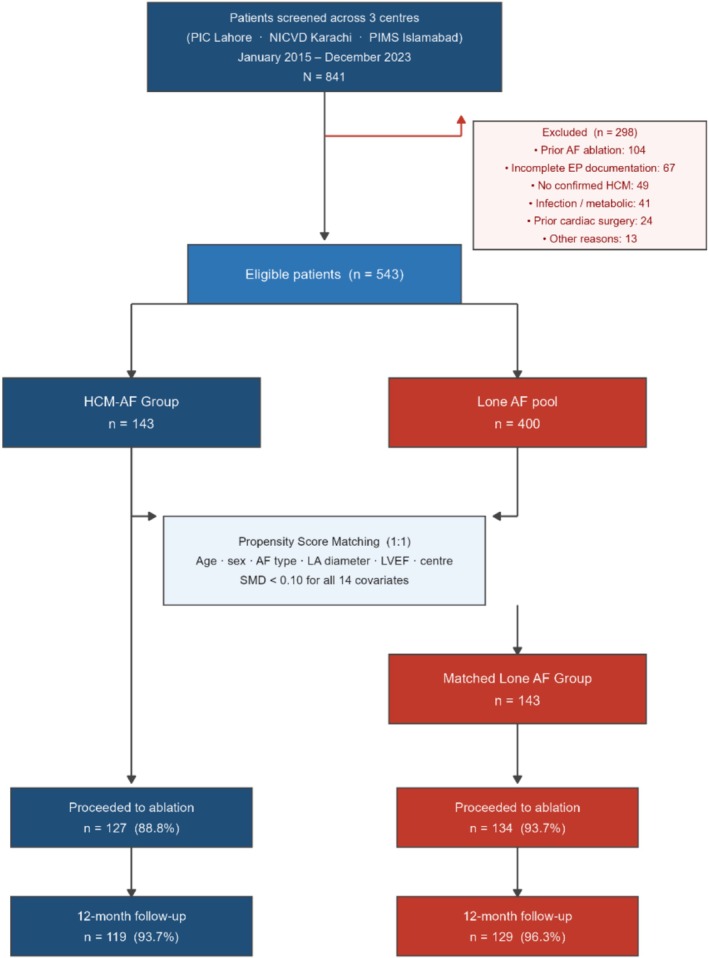
Patient enrolment and follow‐up. AF, atrial fibrillation; HCM, hypertrophic cardiomyopathy; PSM, propensity score matching.

A total of 841 consecutive patients were screened across the three participating centers between January 2015 and December 2023. Of these, 298 were excluded: 104 for prior AF ablation, 67 for incomplete EP documentation, 49 for unconfirmed HCM, 41 for active infection or metabolic disturbance, 24 for prior cardiac surgery, and 13 for other reasons (Figure 1). Of the 543 eligible patients, 143 fulfilled HCM‐AF criteria. From the remaining 400 lone AF patients, 143 were matched 1:1 by propensity score, yielding a final cohort of 286 patients.

**FIGURE 2:**
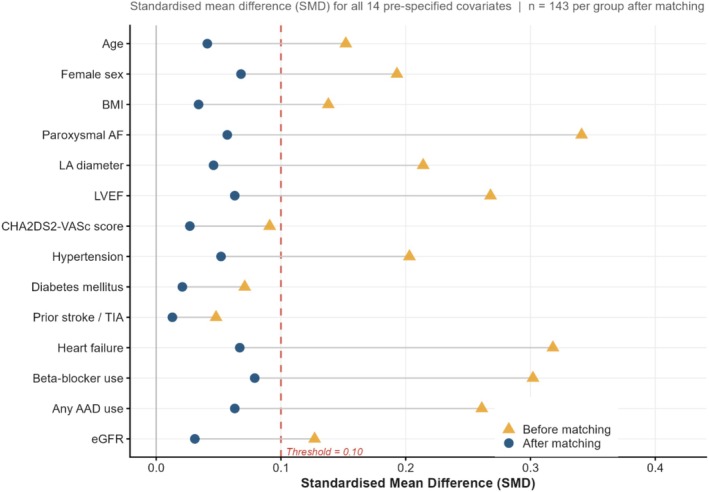
Covariate balance before and after propensity score matching. Dashed line = balance threshold of 0.10. SMD, standardized mean difference.

Baseline characteristics are presented in Table 1. After matching, all 14 covariates achieved SMD < 0.10 (Figure 2). Among HCM‐AF patients, 89 (62.2%) had obstructive HCM (LVOT gradient ≥ 30 mmHg), 63 (44.1%) had at least moderate mitral regurgitation, and 33 (23.1%) carried a preexisting ICD.

**TABLE 1:** Baseline characteristics of the propensity‐score‐matched cohort.

Variable	HCM‐AF (*n* = 143)	Lone AF (*n* = 143)	SMD
Age (years), mean ± SD	51.7 ± 12.3	53.4 ± 11.1	0.07
Female sex, *n* (%)	57 (39.9)	55 (38.5)	0.03
BMI (kg/m^2^), mean ± SD	28.4 ± 4.8	28.1 ± 4.5	0.06
Hypertension, *n* (%)	71 (49.7)	68 (47.6)	0.04
Diabetes mellitus, *n* (%)	34 (23.8)	31 (21.7)	0.05
CHA_2_DS_2_‐VASc, mean ± SD	2.4 ± 1.3	2.3 ± 1.2	0.08
HF hospitalization, *n* (%)	28 (19.6)	25 (17.5)	0.05
CKD (eGFR < 60), *n* (%)	12 (8.4)	10 (7.0)	0.05
AAD use at baseline, *n* (%)	98 (68.5)	94 (65.7)	0.06
AF type—non‐PAF, *n* (%)	106 (74.1)	103 (72.0)	0.05
LA diameter (mm), mean ± SD	44.2 ± 6.7	43.5 ± 6.1	0.05
LAVI (mL/m^2^), mean ± SD	49.6 ± 15.1	48.2 ± 14.3	0.05
LVEF (%), mean ± SD	61.3 ± 8.4	62.1 ± 7.9	0.05
Recruiting center	—	—	0.04
E/e′ ratio^a^	15.4 ± 5.6	10.1 ± 3.7	— (*p* < 0.001)

*Note:* SMD < 0.10 for all 14 propensity‐score‐matched covariates.

Abbreviations: AAD, antiarrhythmic drug; AF, atrial fibrillation; BMI, body mass index; CKD, chronic kidney disease; HF, heart failure; LA, left atrium; LAVI, left atrial volume index; LVEF, left ventricular ejection fraction; PAF, paroxysmal atrial fibrillation; SD, standard deviation; SMD, standardized mean difference.

^a^ E/e′ ratio was not included in the propensity score model (see Section 2.2) and is compared using Student's *t*‐test.

### 3.2 Electrophysiological Characteristics

Intracardiac EP variables are summarized in Table 2. Sinus node function was more frequently impaired in HCM‐AF patients, with significantly prolonged corrected SNRT and AH interval. AERP was uniformly prolonged across all three measurement sites, with markedly greater per‐patient spatial dispersion (41.3 ± 15.7 ms vs. 18.6 ± 9.4 ms, *p* < 0.001). Intra‐atrial conduction was significantly impaired, with P‐wave duration prolonged by 18.2 ms (*p* < 0.001) and CS activation time extended by 19.1 ms (*p* < 0.001). AF was inducible in 117 of 143 HCM‐AF patients (81.8%) versus 84 of 143 lone AF patients (58.7%; RR 1.39, 95% CI 1.18–1.65, *p* < 0.001), with a shorter threshold pacing cycle length (*p* < 0.001).

**TABLE 2:** Intracardiac electrophysiological variables.

Variable	HCM‐AF (*n* = 143)	Lone AF (*n* = 143)	*p*
Corrected SNRT (ms)	391.7 ± 88.4	338.2 ± 69.6	< 0.001
AH interval (ms)	98.3 ± 24.1	87.6 ± 19.3	< 0.001
AERP—HRA (ms)	251.4 ± 33.7	219.8 ± 27.4	< 0.001
AERP—CS proximal (ms)	244.8 ± 36.1	216.7 ± 26.9	< 0.001
AERP—CS distal (ms)	241.3 ± 34.4	213.2 ± 25.6	< 0.001
AERP dispersion (ms)	41.3 ± 15.7	18.6 ± 9.4	< 0.001
Total P‐wave duration (ms)	141.3 ± 19.4	123.1 ± 13.8	< 0.001
CS activation time (ms)	94.7 ± 23.8	75.6 ± 17.9	< 0.001
AF inducibility, *n* (%)	117 (81.8)	84 (58.7)	< 0.001
Threshold CL for induction (ms)	236.8 ± 33.4	263.9 ± 37.7	< 0.001
LA LVA burden (% surface)	29.7 ± 17.4	11.8 ± 9.7	< 0.001
Dense scar < 0.1 mV (% surface)	7.3 ± 5.8	1.3 ± 1.7	< 0.001
CFAE burden (% surface)	15.2 ± 9.1	5.9 ± 4.7	< 0.001
Posterior wall LVA, *n* (%)	102 (71.3)	35 (24.5)	< 0.001
Septal wall LVA, *n* (%)	85 (59.4)	23 (16.1)	< 0.001
Anterior wall LVA, *n* (%)	38 (26.6)	49 (34.3)	0.14
PV–LA conduction slowing, *n* (%)	28 (19.6)	11 (7.7)	0.003
Spontaneous PV ectopy, *n* (%)	41 (28.7)	62 (43.4)	0.008

*Note:* Values are mean ± SD or *n* (%). AERP dispersion was calculated per patient as maximum minus minimum AERP across three sites.

Abbreviations: AERP, atrial effective refractory period; CFAE, complex fractionated atrial electrograms; CL, cycle length; CS, coronary sinus; HRA, high right atrium; LA, left atrium; LVA, low‐voltage area; PV, pulmonary vein; SNRT, sinus node recovery time.

**FIGURE 3:**
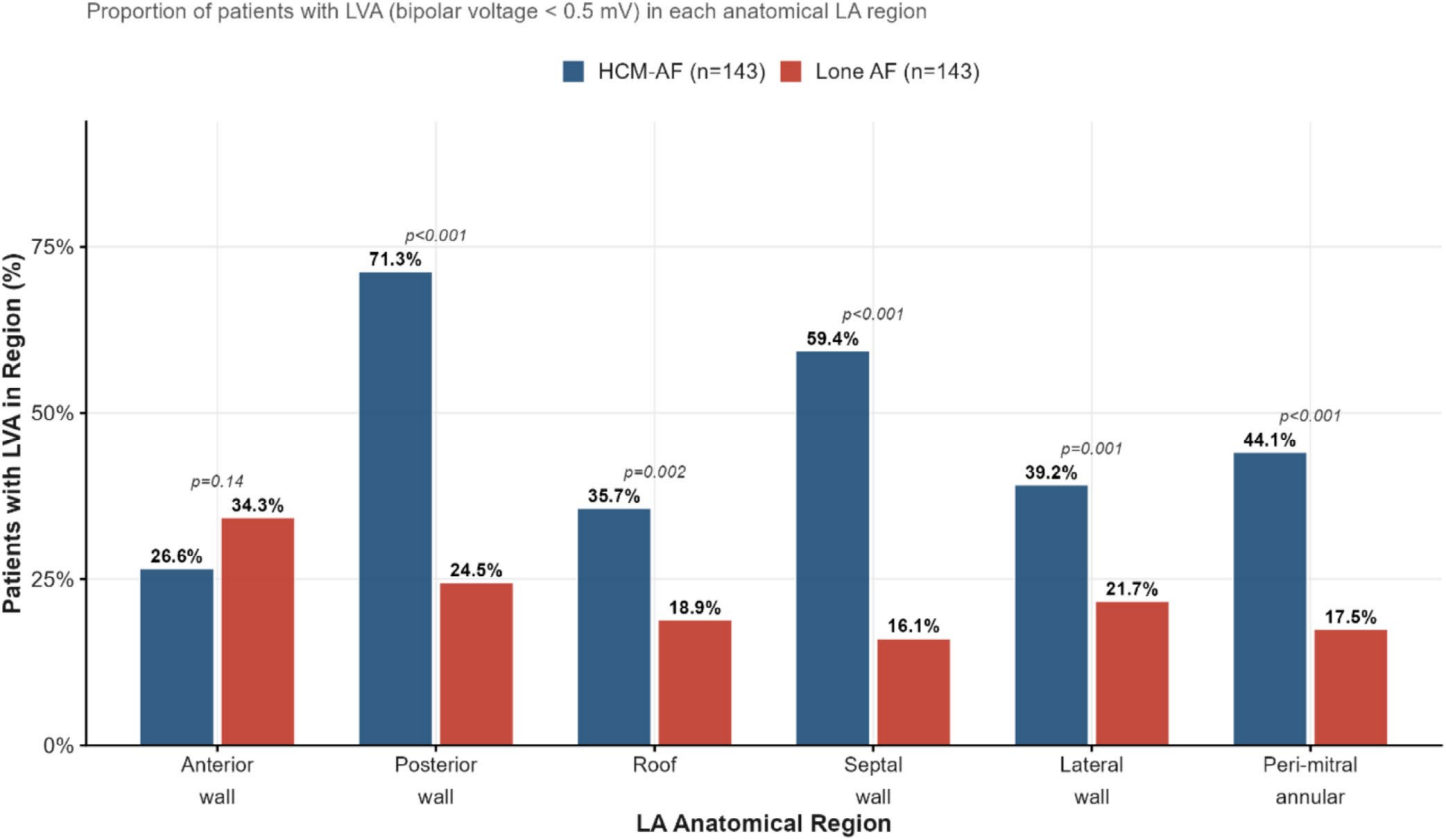
Regional distribution of left atrial low‐voltage area. HCM, hypertrophic cardiomyopathy; LA, left atrium; LVA, low‐voltage area.

LA voltage mapping was completed in all 286 patients. Mean LVA burden was more than twice as high in HCM‐AF (29.7% ± 17.4% vs. 11.8% ± 9.7%, mean difference 17.9%, 95% CI 14.7%–21.1%, *p* < 0.001). Dense scar was significantly greater in HCM‐AF (7.3% ± 5.8% vs. 1.3% ± 1.7%, *p* < 0.001). Regional LVA distribution differed substantially (Figure 3): posterior wall and septal LVA were each significantly more prevalent in HCM‐AF (both *p* < 0.001), whereas anterior wall LVA did not differ (*p* = 0.14). CFAE burden was greater in HCM‐AF, while spontaneous PV ectopy was more frequent in lone AF controls (Table 2).

### 3.3 Ablation Procedural Characteristics and Acute Outcomes

Of 286 matched patients, 261 (91.3%) proceeded to ablation: 127 HCM‐AF (88.8%) and 134 lone AF (93.7%, *p* = 0.13). Procedural details are presented in Table 3. Procedure time, fluoroscopy time, and LA dwell time were all significantly longer in HCM‐AF. Additional ablation beyond PVI was performed in 71.7% of HCM‐AF versus 41.8% of lone AF patients (*p* < 0.001), with the most common targets being posterior wall isolation (51.2%), mitral isthmus line (39.4%), roof line (31.5%), and CFAE‐guided ablation (21.3% vs. 4.5%, *p* < 0.001). Acute PVI success was achieved in 89.8% of HCM‐AF versus 95.5% of lone AF patients (*p* = 0.09). DC cardioversion was required more frequently in HCM‐AF (25.2% vs. 9.7%, *p* < 0.001). Major complication rates were low and comparable between groups (4.7% vs. 2.2%, *p* = 0.33). Median hospital stay was longer in HCM‐AF (3 vs. 2 days, *p* = 0.001).

**TABLE 3:** Ablation procedural characteristics and acute outcomes.

Variable	HCM‐AF (*n* = 127)	Lone AF (*n* = 134)	*p*
Total procedure time (min)	193.6 ± 52.4	137.8 ± 38.1	< 0.001
Total fluoroscopy time (min)	43.7 ± 19.2	27.6 ± 11.8	< 0.001
LA dwell time (min)	131.2 ± 41.7	89.4 ± 28.3	< 0.001
PVI performed, *n* (%)	127 (100)	134 (100)	—
Acute PVI success, *n* (%)	114 (89.8)	128 (95.5)	0.09
Additional ablation, *n* (%)	91 (71.7)	56 (41.8)	< 0.001
Posterior wall isolation	65 (51.2)	19 (14.2)	< 0.001
Mitral isthmus line	50 (39.4)	14 (10.4)	< 0.001
Roof line	40 (31.5)	12 (9.0)	< 0.001
CFAE‐guided ablation	27 (21.3)	6 (4.5)	< 0.001
DC cardioversion required, *n* (%)	32 (25.2)	13 (9.7)	< 0.001
Major complications, *n* (%)	6 (4.7)	3 (2.2)	0.33^a^
Hospital stay (days), median	3	2	0.001

*Note:* Values are mean ± SD, *n* (%), or median.

Abbreviations: CFAE, complex fractionated atrial electrograms; DC, direct current; LA, left atrium; PVI, pulmonary vein isolation.

^a^ Fisher's exact test.

### 3.4 Primary and Secondary Outcomes at 12 Months

**FIGURE 4:**
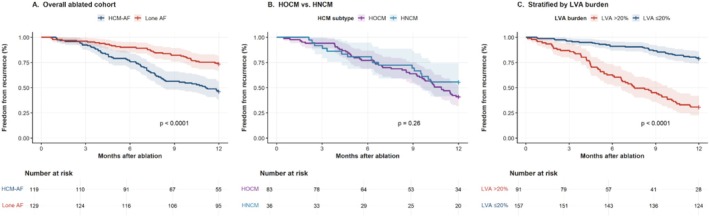
Kaplan–Meier estimates of 12‐month freedom from atrial arrhythmia recurrence. HNCM, nonobstructive HCM; HOCM, obstructive HCM; LVA, low‐voltage area.

Twelve‐month follow‐up was available for 248 of 261 ablated patients (95.0%): 119 HCM‐AF (93.7%) and 129 lone AF (96.3%). Outcomes are presented in Table S1 and Figure 4.

The KM estimate for 12‐month freedom from atrial arrhythmia recurrence was 51.7% (95% CI 42.3%–60.6%) in HCM‐AF versus 73.9% (95% CI 65.7%–80.7%) in lone AF (log‐rank *p* = 0.001; Figure 4, panel A). After multivariable adjustment, HCM diagnosis remained an independent predictor of recurrence (aHR 2.19, 95% CI 1.47–3.26, *p* < 0.001). Obstructive HCM was associated with worse recurrence‐free survival than nonobstructive HCM (46.3% vs. 61.8%, log‐rank *p* = 0.032; Figure 4, panel B). Patients with LVA burden > 20% demonstrated markedly inferior outcomes regardless of group (38.4% vs. 76.1%, log‐rank *p* < 0.001; Figure 4, panel C). Notably, 14 of 119 HCM‐AF patients (11.8%) experienced sustained ventricular arrhythmias during follow‐up, compared with none in lone AF (Fisher's exact test, *p* < 0.001).

### 3.5 Multivariable Predictors and Advanced Analyses

Cox regression results are shown in Table S2; model performance in Figure S1. Four variables independently predicted recurrence: LVA burden (aHR 1.41 per 10% increase, *p* < 0.001), HCM diagnosis (aHR 2.19, *p* < 0.001), non‐paroxysmal AF (aHR 1.88, *p* = 0.003), and AERP dispersion ≥ 30 ms (aHR 1.67, *p* = 0.019). Harrell's C‐statistic was 0.74 (95% CI 0.67–0.81).

Latent class analysis of 14 EP substrate variables identified three distinct phenotype clusters across the 286 matched patients (Figure S2). Cluster 1 (*n* = 79, 27.6%) was characterized by minimal LVA burden (mean 7.3%) and high PV trigger dependence, with 97.5% from the lone AF group. Cluster 2 (*n* = 113, 39.5%) exhibited intermediate substrate with mixed group composition (54.0% HCM‐AF). Cluster 3 (*n* = 94, 32.9%) showed extensive LVA burden (mean 41.6%), posterior‐wall‐predominant scar, and high CFAE density, with 85.1% from the HCM‐AF group. Freedom from recurrence differed dramatically: 81.7% (Cluster 1), 61.4% (Cluster 2), and 31.9% (Cluster 3; log‐rank *p* < 0.001). The solution was robust across 500 bootstrap iterations (silhouette coefficient 0.61). Subgroup analyses confirmed consistent excess recurrence risk in HCM‐AF, with significant interactions for AF type, LVA burden, and AERP dispersion (Figure S3).

## 4 Discussion

In this multicenter propensity‐score‐matched study, we comprehensively characterized the intracardiac electrophysiological substrate of AF in HCM and compared it with matched lone AF controls. Three principal findings emerged. First, HCM‐AF patients exhibited a profoundly more arrhythmogenic left atrial substrate, with substantially greater low‐voltage area burden, wider spatial dispersion of atrial refractoriness, prolonged conduction, and higher AF inducibility. Second, despite more frequent additional ablation beyond PVI, 12‐month freedom from recurrence was significantly lower in HCM‐AF, and HCM diagnosis remained an independent predictor of recurrence. Third, latent class analysis identified three distinct EP substrate phenotypes with dramatically divergent outcomes, suggesting that data‐driven substrate characterization may enable clinically meaningful patient stratification.

The greater left atrial fibrotic burden and refractoriness dispersion observed in HCM‐AF patients are consistent with the known pathophysiology of the disease. Diffuse interstitial and replacement fibrosis—driven by sarcomeric dysfunction, myocyte disarray, and microvascular ischaemia—extends beyond the ventricular myocardium into the atrial walls [18, 19], creating a heterogeneous substrate characterized by slow conduction, increased anisotropy, and regional refractoriness heterogeneity [20]. The predominantly posterior and septal distribution of LVA in our HCM‐AF cohort, distinct from the anterior wall predominance sometimes described in lone AF, suggests disease‐specific pathological mechanisms rather than simple haemodynamic left atrial stretch.

The inferior ablation outcomes in HCM‐AF patients—despite more extensive substrate modification—suggest that the complexity of the atrial myopathy in HCM exceeds what current ablation strategies can adequately address. This aligns with the concept that PVI alone is insufficient in extensive extra‐PV substrate, yet aggressive substrate modification does not fully compensate for diffuse myopathic remodeling [6, 21]. The strong independent association between LVA burden and recurrence irrespective of group reinforces LVA burden as a determinant of ablation failure that transcends disease etiology. The occurrence of sustained ventricular arrhythmias exclusively in the HCM‐AF group was an unexpected but clinically important observation, underscoring that these patients carry a broader arrhythmic vulnerability beyond AF [22].

Our finding that HCM‐AF patients harbor more extensive left atrial fibrosis than non‐HCM controls is concordant with Efremidis et al. [8], who demonstrated wider fibrotic regions in propensity‐matched HCM patients and identified LVA burden as the sole predictor of recurrence, and is further supported by evidence that HCM‐specific sarcomeric mutations may independently promote atrial fibrosis [23]. However, their study included only 28 patients from a single‐center; our larger multicenter cohort strengthens the generalisability of this observation.

The single‐procedure recurrence rate in our HCM‐AF cohort is consistent with existing data. Dinshaw et al. [12] reported long‐term freedom from atrial arrhythmias in 60% of 65 HCM patients after a mean of 1.9 ablation procedures, with a subsequent meta‐analysis by the same group [10] demonstrating a pooled relative risk of 1.50 for recurrence in HCM versus non‐HCM. An earlier meta‐analysis by Providencia et al. [24] and a proportional meta‐analysis by Ahmed et al. [9] similarly reported single‐procedure success rates of only 39%–52% in HCM, while Lin et al. [25] confirmed comparable findings in an Asian HCM‐AF cohort. Müssigbrodt et al. [23] further showed inferior ablation outcomes in patients with hypertrophied hearts regardless of the underlying etiology. Our results extend these data by demonstrating that the excess recurrence risk in HCM is attributable to quantifiable substrate differences, particularly LVA burden and AERP dispersion, rather than HCM diagnosis per se.

Regarding additional ablation beyond PVI, the ERASE‐AF trial demonstrated that individualized LVA‐guided substrate modification significantly reduced recurrence in persistent AF [26], whereas the SUPPRESS‐AF trial found no significant benefit [27]. In HCM specifically, Rowin et al. [21] and Santangeli et al. [22] highlighted that non‐PV triggers may represent the dominant mechanism for recurrence. Our data support this: despite extensive additional ablation, patients with the highest substrate burden continued to have poor outcomes, suggesting that the diffuse HCM atrial myopathy extends beyond what focal or linear strategies can address.

The safety profile was reassuring. Major complication rates were low and comparable between groups, with no procedure‐related deaths, consistent with the Cryo Global Registry [28] and the meta‐analyses by Dinshaw et al. [10] and Providencia et al. [24], confirming that ablation in HCM does not carry disproportionately higher procedural risk at experienced centers.

### 4.1 Clinical Implications

The identification of LVA burden as the strongest independent predictor of recurrence suggests that pre‐ablation voltage mapping should be routine in HCM‐AF patients. The divergent recurrence rates across EP phenotype clusters indicate that a one‐size‐fits‐all approach is suboptimal; patients with extensive substrate may benefit from intensified surveillance, lower thresholds for repeat ablation, or novel cardiac myosin inhibitors such as mavacamten added to standard therapy, which significantly reduce hospitalizations, emergency visits, hemodynamic instability, and improve left ventricular ejection fraction [29, 30]. The ventricular arrhythmia burden observed exclusively in the HCM‐AF group further underscores the need for comprehensive arrhythmia risk assessment and consideration of implantable cardioverter‐defibrillator therapy, which demonstrates a favorable risk–benefit profile with an annualized appropriate intervention rate of 4.0% and low all‐cause mortality of 1.3% per year in HCM patients [31]. Recent evidence also highlights important limitations of temporary sinus rhythm restoration strategies before catheter ablation in longstanding persistent atrial fibrillation, reinforcing the importance of substrate‐guided approaches over reliance on pre‐procedural rhythm control [32].

### 4.2 Limitations

The retrospective design introduces potential selection bias in ablation candidacy and operator‐dependent target selection. Intermittent ECG and 24‐h Holter monitoring likely underestimated recurrence in both groups [33]. The 12‐month follow‐up may miss late recurrences known to occur in HCM [12]. Generalisability to other ethnic populations requires confirmation given potential differences in HCM phenotype distribution.

### 4.3 Future Directions

Prospective studies with continuous monitoring (implantable loop recorders) are needed to define true recurrence rates and validate the EP phenotype clusters as prognostic tools. The role of CMR‐derived left atrial late gadolinium enhancement as a complementary substrate characterization tool should be explored [34]. Whether pulsed‐field ablation improves outcomes in the thickened HCM atrium warrants investigation. Finally, the potential for cardiac myosin inhibitors to modify the atrial substrate represents a compelling translational research avenue.

## 5 Conclusion

HCM‐AF was associated with a profoundly more arrhythmogenic atrial substrate compared with lone AF, and catheter ablation outcomes were significantly inferior despite more extensive intervention. LVA burden, HCM diagnosis, non‐paroxysmal AF, and AERP dispersion independently predicted recurrence. Three distinct EP phenotype clusters with divergent outcomes support a substrate‐stratified approach to ablation planning in this challenging population.

## Author Contributions

**Umair Abrar:** conceptualization, methodology, data curation, formal analysis, writing – original draft. **Fazal Ghaffar:** data curation, investigation, writing – review and editing. **Muhammad Zuhaid:** data curation, investigation, writing – review and editing. **Huzaifa Bin Afzaal:** formal analysis, data curation, writing – review and editing. **Palwasha Abbasi:** data curation, investigation, writing – review and editing. **Muhammad Hasnain Iqbal:** data curation, investigation, writing – review and editing. **Moʻminov Jahongir Zokirjon oʻgʻli:** data curation, writing – review and editing. **Sifat Ullah Safi:** conceptualization, supervision, project administration, writing – review and editing. All authors read and approved the final manuscript.

## Funding

The authors have nothing to report.

## Ethics Statement

This study was approved by the Ethics Review Committee of the National Institute of Cardiovascular Diseases, Karachi (NICVD/IRB/2023‐312), the Institutional Review Board of the Punjab Institute of Cardiology, Lahore (PIC/ERC/2023‐847), and the Ethics Committee of the Pakistan Institute of Medical Sciences, Islamabad (PIMS/EC/2024‐0158). The study was conducted in accordance with the principles outlined in the Declaration of Helsinki. Given the retrospective, observational, and non‐interventional nature of the study, a waiver of individual written informed consent was granted by each institutional review board. All patient‐identifying information was removed prior to data extraction and analysis to ensure confidentiality.

## Consent

A waiver of individual written informed consent was approved by the institutional review board of each participating center due to the retrospective, observational, and non‐interventional design of the study. All patient data were anonymized prior to analysis.

## Conflicts of Interest

The authors declare no conflicts of interest.

## Supporting information


**Figure S1:** Model discrimination and calibration.
**Figure S2:** Latent EP phenotype cluster analysis.
**Figure S3:** Subgroup forest plot—AF recurrence risk: HCM‐AF versus lone AF.
**Table S1:** Primary and secondary outcomes at 12 months.
**Table S2:** Cox proportional hazards analysis for 12‐month AF recurrence.

## Data Availability

The data that support the findings of this study are available on request from the corresponding author. The data are not publicly available due to privacy or ethical restrictions.
