# Supplementary material for: Electrophysiological Substrate and Ablation Outcomes in Atrial Fibrillation With Hypertrophic Cardiomyopathy: A Propensity‐Matched Multicenter Study
Source: J Arrhythm. 2026 May 23;42(3):e70364. doi: 10.1002/joa3.70364 (PMC13240565; doi:10.1002/joa3.70364)
Supplement: Supplementary file 1 — Figure S1: Model discrimination and calibration. Figure S2: Latent EP phenotype cluster analysis. Figure S3: Subgroup forest plot—AF recurrence risk: HCM‐AF versus lone AF. Table S1: Primary and secondary outcomes at 12 months. Table S2: Cox proportional hazards analysis for 12‐month AF recurrence. [file JOA3-42-e70364-s001.docx]

**SUPPLEMENTARY FILE**

**Supplementary Figure S1.** Model Discrimination and Calibration.

**Supplementary Figure S2.** Latent EP Phenotype Cluster Analysis.

**Supplementary Figure S3.** Subgroup Forest Plot — AF Recurrence Risk: HCM-AF versus

Lone AF.

**Supplementary Figure S4. Covariate balance in the ablated subset after propensity-score matching (n=261).**

**Supplementary Table S1.** Primary and Secondary Outcomes at 12 Months

**Supplementary Table S2.** Cox Proportional Hazards Analysis for 12-Month AF Recurrence

**Supplementary Table S3**. Baseline Characteristics of the Ablated Subset (n=261)

**Supplementary Figure S1. Model Discrimination and Calibration.**


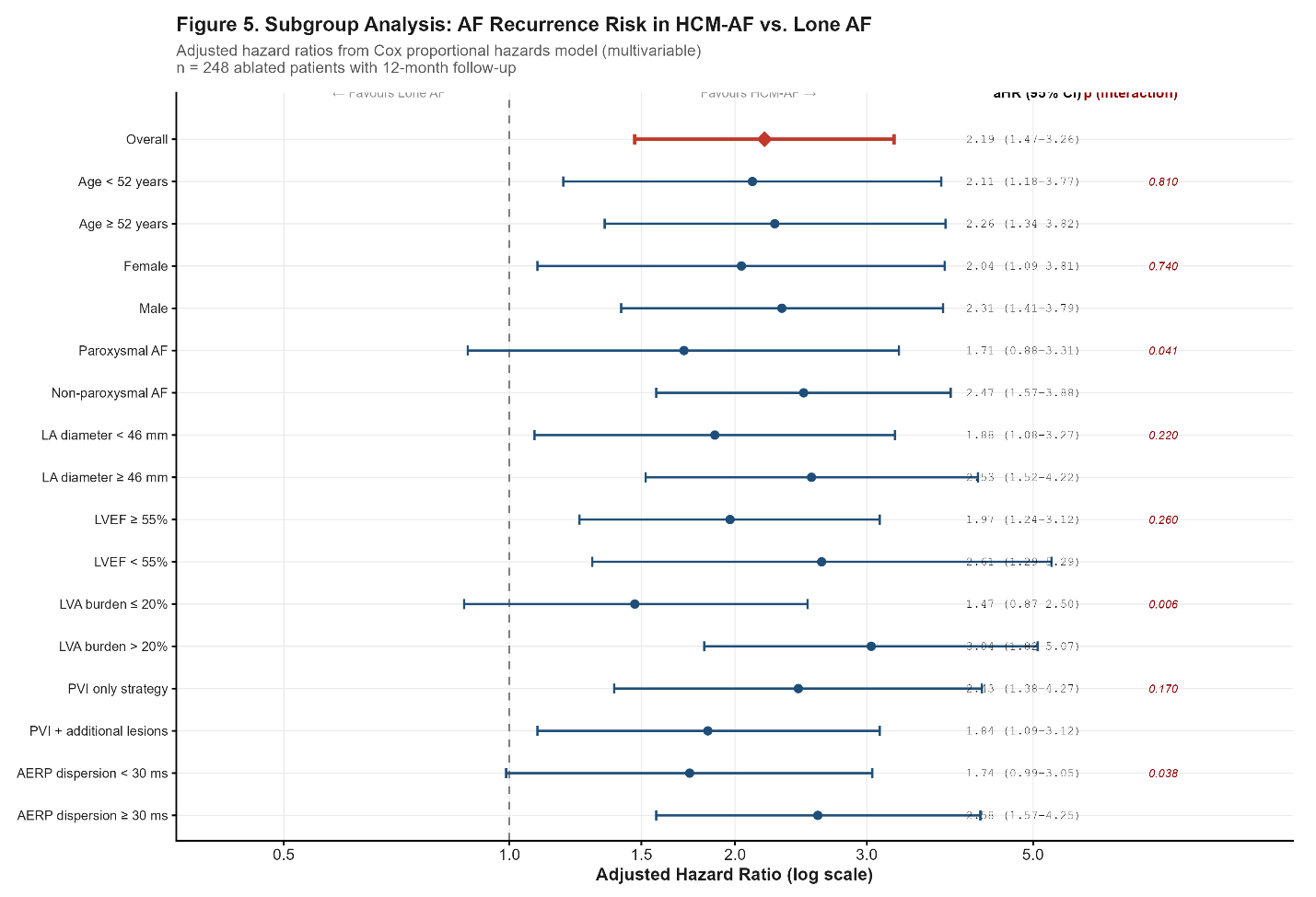


Abbreviations: *CI, confidence interval.*

**Supplementary Figure S2. Latent EP Phenotype Cluster Analysis.**


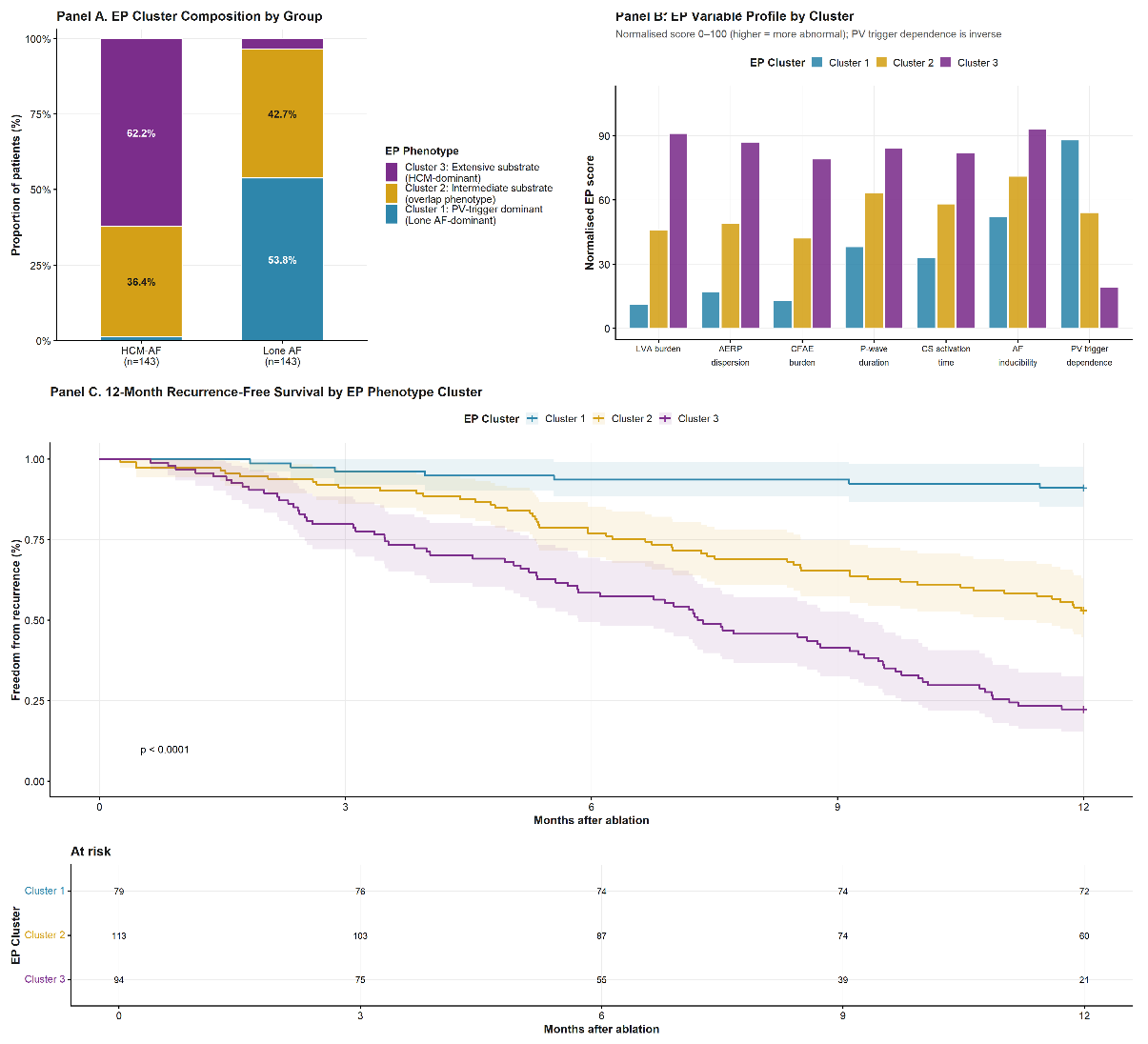


*Abbreviations: AERP, atrial effective refractory period; EP, electrophysiology; LVA, low-voltage area.*

**Supplementary Figure S3. Subgroup Forest Plot — AF Recurrence Risk: HCM-AF versus Lone AF**


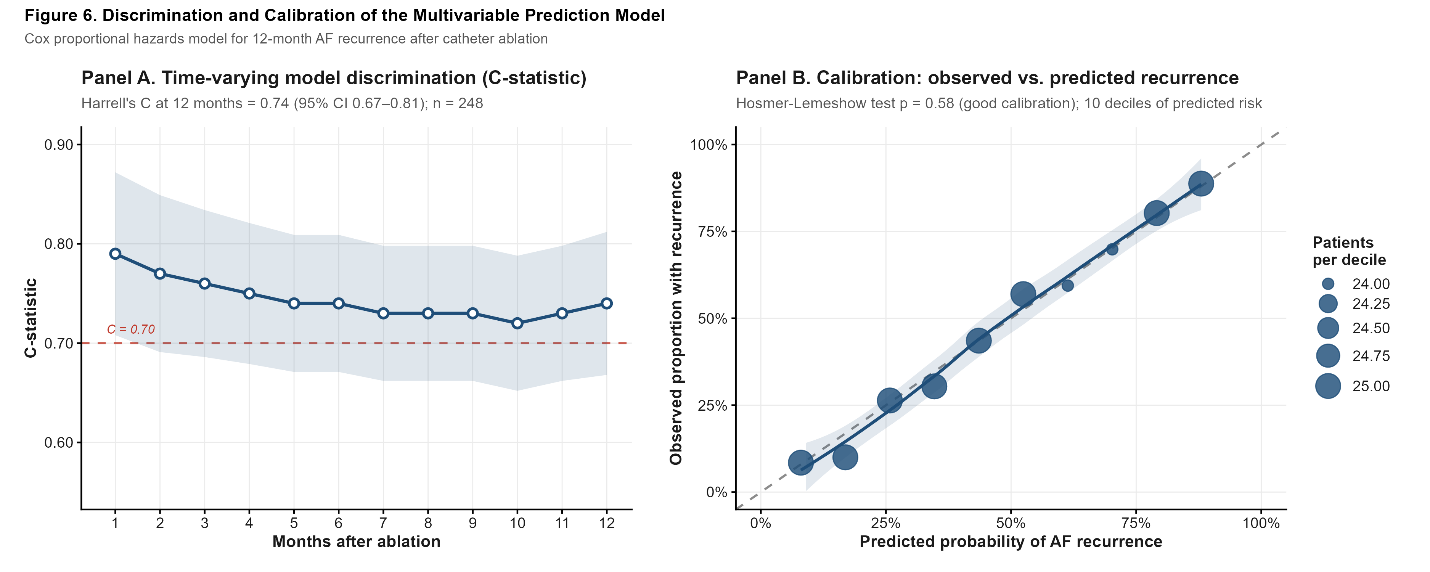


*AERP, atrial effective refractory period; EP, electrophysiology; LVA, low-voltage area.*

**Supplementary Figure S4. Covariate balance in the ablated subset after propensity-score matching (n=261).**

*
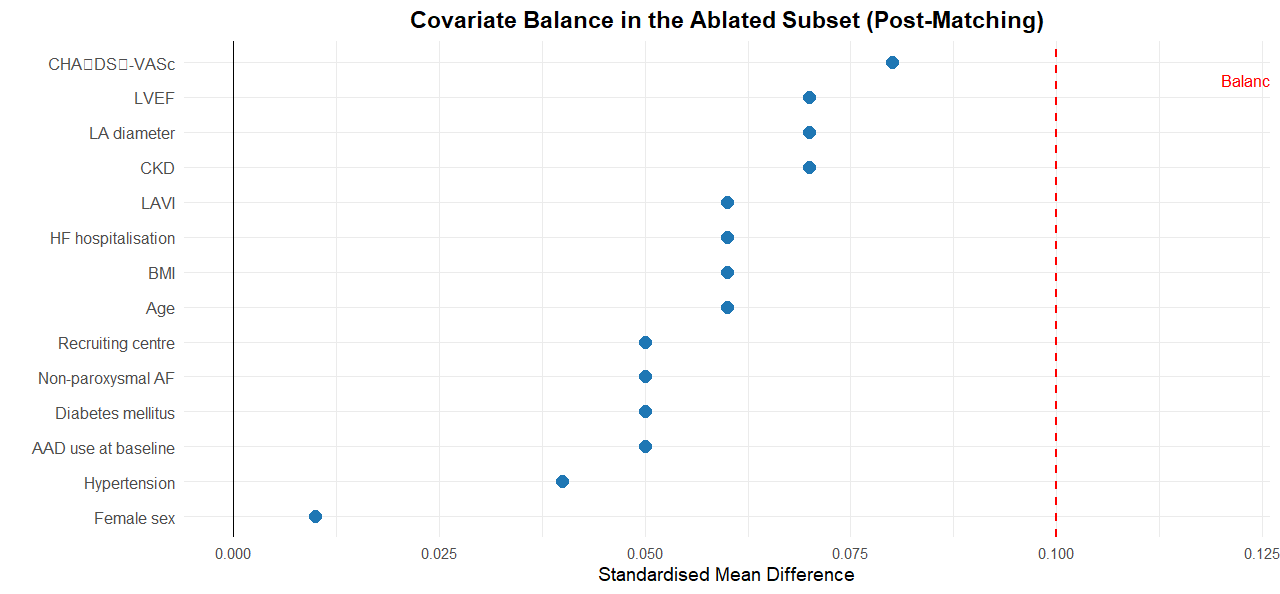
*

*Dashed red line = balance threshold of 0.10. SMD, standardised mean difference.*

**Supplementary Table S1. Primary and Secondary Outcomes at 12 Months**

| **Outcome** | **HCM-AF** | **Lone AF** | **p-value** |
| --- | --- | --- | --- |
| **Primary endpoints** |  |  |  |
| LA LVA burden (%), mean±SD | 29.7±17.4 | 11.8±9.7 | <0.001 |
| AF inducibility, n/N (%) | 117/143 (81.8) | 84/143 (58.7) | <0.001 |
| 12-mo freedom from recurrence, % (95% CI) | 51.7 (42.3–60.6) | 73.9 (65.7–80.7) | 0.001ᵃ |
| **Secondary endpoints** |  |  |  |
| Acute PVI success, n/N (%) | 114/127 (89.8) | 128/134 (95.5) | 0.09 |
| Major complication, n/N (%) | 6/127 (4.7) | 3/134 (2.2) | 0.33ᵇ |
| AAD use at 12 months, n/N (%) | 72/119 (60.5) | 41/129 (31.8) | <0.001 |
| Sustained VA, n/N (%) | 14/119 (11.8) | 0/129 (0) | <0.001ᵇ |
| All-cause mortality, n/N (%) | 2/127 (1.6) | 0/134 (0) | 0.23ᵇ |

*ᵃLog-rank test. ᵇFisher's exact test. AF recurrence defined as any documented AF/AT/AFL episode >30s after the 90-day blanking period. 12-month follow-up available in 119/127 HCM-AF and 129/134 lone AF ablated patients. Abbreviations: AAD, antiarrhythmic drug; AF, atrial fibrillation; AFL, atrial flutter; AT, atrial tachycardia; CI, confidence interval; LA, left atrium; LVA, low-voltage area; PVI, pulmonary vein isolation; VA, ventricular arrhythmia*

**Supplementary Table S2. Cox Proportional Hazards Analysis for 12-Month AF Recurrence**

| **Variable** | **Univariable HR (95% CI)** | **p-value** | **Multivariable aHR (95% CI)** | **p-value** |
| --- | --- | --- | --- | --- |
| HCM diagnosis | 2.41 (1.64–3.54) | <0.001 | 2.19 (1.47–3.26) | <0.001 |
| LA LVA burden (per 10% ↑) | 1.49 (1.30–1.71) | <0.001 | 1.41 (1.22–1.63) | <0.001 |
| Non-paroxysmal AF type | 2.03 (1.38–2.99) | <0.001 | 1.88 (1.24–2.86) | 0.003 |
| AERP dispersion ≥30ms | 1.84 (1.22–2.77) | 0.004 | 1.67 (1.09–2.57) | 0.019 |
| LAVI (per 10 mL/m² ↑) | 1.21 (1.04–1.41) | 0.014 | NS | — |
| LA diameter (per 5mm ↑) | 1.18 (1.02–1.37) | 0.028 | NS | — |
| LVEF <50% | 1.72 (0.98–3.02) | 0.058 | NS | — |
| E/e' ratio >14 | 1.61 (1.08–2.40) | 0.019 | NS | — |
| No additional lesions beyond PVI | 1.53 (1.03–2.27) | 0.036 | NS | — |
| CFAE burden (per 5% ↑) | 1.19 (1.02–1.39) | 0.029 | NS | — |
| CHA₂DS₂-VASc score | 1.14 (0.98–1.33) | 0.09 | NS | — |

*Propensity-score-matched ablated cohort (n=248 with 12-month follow-up). Variables meeting p<0.10 on univariable analysis entered into multivariable model. Harrell's C-statistic: 0.74 (95% CI 0.67–0.81). Proportional hazards assumption verified by Schoenfeld residuals (all p>0.12). NS, not significant in multivariable model. Abbreviations: AERP, atrial effective refractory period; aHR, adjusted hazard ratio; CFAE, complex fractionated atrial electrograms; CI, confidence interval; HCM, hypertrophic cardiomyopathy; HR, hazard ratio; LA, left atrium; LAVI, left atrial volume index; LVA, low-voltage area; LVEF, left ventricular ejection fraction; PVI, pulmonary vein isolation.*

**Supplementary Table S3. Baseline Characteristics of the Ablated Subset (n=261)**

| **Variable** | **HCM-AF (n=127)** | **Lone AF (n=134)** | **SMD** |
| --- | --- | --- | --- |
| Age (years), mean±SD | 51.9±12.1 | 53.1±11.0 | 0.06 |
| Female sex, n (%) | 50 (39.4) | 52 (38.8) | 0.01 |
| BMI (kg/m²), mean±SD | 28.5±4.7 | 28.0±4.4 | 0.06 |
| Hypertension, n (%) | 63 (49.6) | 64 (47.8) | 0.04 |
| Diabetes mellitus, n (%) | 30 (23.6) | 29 (21.6) | 0.05 |
| CHA₂DS₂-VASc, mean±SD | 2.4±1.3 | 2.3±1.2 | 0.08 |
| HF hospitalisation, n (%) | 25 (19.7) | 23 (17.2) | 0.06 |
| CKD (eGFR<60), n (%) | 11 (8.7) | 9 (6.7) | 0.07 |
| AAD use at baseline, n (%) | 87 (68.5) | 89 (66.4) | 0.05 |
| AF type — non-PAF, n (%) | 95 (74.8) | 97 (72.4) | 0.05 |
| LA diameter (mm), mean±SD | 44.3±6.6 | 43.4±6.0 | 0.07 |
| LAVI (mL/m²), mean±SD | 49.8±15.0 | 48.0±14.1 | 0.06 |
| LVEF (%), mean±SD | 61.1±8.5 | 62.3±7.8 | 0.07 |
| Recruiting centre | — | — | 0.05 |
